# Developing LHS scholars’ competency around reducing burnout and moral injury

**DOI:** 10.1002/lrh2.10378

**Published:** 2023-06-18

**Authors:** Sirin Yilmaz, Michele LeClaire, Abbie Begnaud, Warren McKinney, Kasey R. Boehmer, Cory Schaffhausen, Mark Linzer

**Affiliations:** ^1^ Clinical Ethics, Hennepin Healthcare Minneapolis Minnesota USA; ^2^ Department of Medicine Minneapolis VA Health Care System Minneapolis Minnesota USA; ^3^ Department of Medicine University of Minnesota Minneapolis Minnesota USA; ^4^ Department of Medicine Hennepin Healthcare Research Institute (HHRI) Minneapolis Minnesota USA; ^5^ Department of Medicine, Division of Health Care Delivery Research and Knowledge and Evaluation Research (KER) Unit Mayo Clinic Rochester Minnesota USA

**Keywords:** burnout, ethical climate, LHS (learning health system), moral injury

## Abstract

Despite the known benefits of supportive work environments for promoting patient quality and safety and healthcare worker retention, there is no clear mandate for improving work environments within Learning Health Systems (LHS) nor an LHS wellness competency. Striking rises in burnout levels among healthcare workers provide urgency for this topic.

**Methods:**

We brought three experts on moral injury, burnout prevention, and ethics to a recurring, interactive LHS training program “Design Shop” session, harnessing scholars’ ideas prior to the meeting. Generally following SQUIRE 2.0 guidelines, we evaluated the prework and discussion via informal content analysis to develop a set of pathways for developing moral injury and burnout prevention programs. Along these lines, we developed a new competency for moral injury and burnout prevention within LHS training programs.

**Results:**

In preparation for the session, scholars differentiated moral injury from burnout, highlighted the profound impact of COVID‐19 on moral injury, and proposed testable interventions to reduce injury. Scholar and expert input was then merged into developing the new competency in moral injury and burnout prevention. In particular, the competency focuses on preparing scholars to (1) demonstrate knowledge of moral injury and burnout, (2) measure burnout, moral injury, and their remediable predictors, (3) use methods for improving burnout, (4) structure training programs with supportive work environments, and (5) embed burnout and moral injury prevention into LHS structures.

**Conclusions:**

Burnout and moral injury prevention have been largely omitted in LHS training. A competency related to burnout and moral injury reduction can potentially bring sustainable work lives for scholars and their colleagues, better incorporation of their science into clinical practice, and better outcomes for patients.

## 1 INTRODUCTION

Learning Health Systems (LHS) have well‐established competencies^1^ for supporting embedded research and encouraging prompt system change based on local findings. LHS researcher core competencies focus upon seven domains: (1) systems science; (2) scientific evidence standards; (3) research methods; (4) informatics; (5) health system research ethics; (6) implementation science; and (7) engagement, leadership, and research management. The Minnesota Learning Health System (MN‐LHS) Center of Excellence within the AHRQ‐PCORI LHS K12 program selects two to three scholars per year to train within the program; scholars are Ph.D. or physician scientists typically 3–5 years into postgraduate careers pursuing LHS science while embedded within their institutions. As a core aspect of training, we established an interactive curriculum format, or “Design Shop,” using a Mayo Clinic model with peer mentoring and new science born via collaborations amongst attendees. Biweekly (every other week) 60–90 min sessions cover topics from LHS competencies and encourage new knowledge creation. Here we report on how a Design Shop session sparked a new focus on burnout and moral injury prevention, with a related LHS competency.

Ethical dilemmas in the workplace may result in “moral injury,” are a source of burnout and distress,^2^, ^3^ and present barriers to ethical work climates.^4^ To address this gap in LHS competencies, we used a self‐study format before a Design Shop to enhance the discussion of “Moral Injury and an Ethical Work Climate.” We now offer a call for an LHS competency in moral injury, burnout prevention, and the creation of an ethical climate for healthcare workers (HCWs) throughout LHS and other institutions.

## 2 QUESTIONS WE SEEK TO ANSWER WITH THIS PAPER ARE

(1) What are the underlying tenets of burnout and moral injury prevention, (2) how can burnout and moral injury prevention support an ethical work climate in LHS science, and (3) what is the potential “value added” for a new competency in moral injury and burnout reduction?

## 3 DEFINITIONS, BACKGROUND, AND RELATING WORK LIFE TO LHS CORE VALUES

Burnout and moral injury are overlapping but distinct constructs.^5^, ^6^ Burnout is emotional exhaustion and depersonalization from work stress,^7^ while moral injury is the result of being forced to do something that one believes is ethically unacceptable.^3^, ^6^ Burnout and stress are in part based upon the demand‐control model of job stress, with stress (and burnout, a long‐term stress reaction) rising in response to work demands or diminishing work control.^8^ The MEMO (Minimizing Error Maximizing Outcome) study supported by AHRQ in 2005–2009^7^ in 119 primary care practices showed that almost half of the clinicians needed more time for visits, 27% were burning out (this number has risen^2^ to 50%), and 30% intended to leave their jobs. Strong relationships linked work environments (control, chaos, time pressure, and culture) to adverse outcomes (stress, burnout, and intent to leave). Patient care quality was lower with adverse work conditions, while quality was higher when culture (such as values congruence with leaders) was favorable.^7^ The AHRQ‐funded Healthy Work Place (HWP) randomized trial found three types of workplace interventions favorably affected burnout and/or satisfaction: workflow redesign, communication improvements between provider groups, and quality improvement initiatives sharing work with team members.^9^ Thus, there is strong evidence that workplace stress relates to adverse outcomes for clinicians and patients, and evidence‐based interventions can reduce burnout.

**FIGURE 1:**
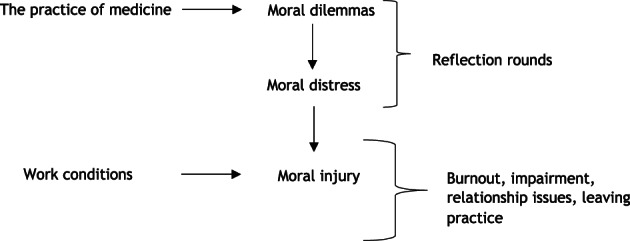
Moral dilemmas leading to moral distress and then moral injury. Based upon work first published in Linzer M, Poplau S. Eliminating burnout and moral injury: Bolder steps required. EClinicalMedicine. 2021 Aug 19;39:101090. doi: 10.1016/j.eclinm.2021.101090. PMID: 34466795; PMCID: PMC8385149.) Reprinted with permission.

In 2019, Dean and colleagues laid the groundwork for discussing moral distress in medicine.^10^ Moral distress, a predecessor of moral injury, occurs when policies or routines conflict with personal beliefs, for example, when clinicians must do things with which they fundamentally disagree.^3^, ^6^, ^10^ LHS scholars might experience moral distress from work overload and system inefficiencies interfering with self‐care and scholarly work; clinicians experience distress when they must give nonbeneficial care to a dying patient. Our conceptual model^11^ shows how moral dilemmas can lead to moral distress (Figure 1), which can lead to moral injury.^12^ Work conditions contribute to these outcomes, while reflection rounds, a powerful tool for debriefing challenging events, can interrupt injurious processes. Epstein's crescendo effect shows how repeated injury episodes lead to lasting impairment.^13^ Compromising one's integrity, a proxy for moral injury, is also linked to adverse outcomes, including burnout and intent to leave the institution,^5^ especially in critical care clinicians and nurses.^5^ Feeling valued by one's organization attenuated adverse outcomes. A brief moral injury metric for clinical use is in development (LeClaire, manuscript in preparation).

The creation of an ethical work climate is an anticipated outcome of reductions in moral injury and burnout.^3^, ^10^, ^11^ This can be important for trainees (eg, not putting ethically meaningful work aside), as well as for clinicians (always acting on behalf of the patient). A *virtues‐based* approach reduces moral injury by balancing classic ethical principles of autonomy, beneficence, nonmaleficence, and justice.^4^, ^14^ Ethicists propose recalibrating the balance between autonomy and beneficence, allowing actions “directed by the good of the patient”.^15^ To link moral injury reduction to more ethical work climates, Savel and Munro^4^ introduce the “four A” framework of Ask (is my team suffering?), Affirm (allow for self‐care), Assess (identify sources of distress) and Act (preserve integrity and authenticity). These questions can identify sources of distress and lead to reduced injury; further studies should test these assertions in clinical environments.

These ethical processes relate to LHS Core Values (www.Learninghealth.org). An LHS aspires to improve the health of individuals and populations, generating and sharing knowledge to allow clinicians to apply best practices and make better decisions. Preventing burnout and moral injury is essential to this process,^16^ as the ability to sustain as an HCW and make good decisions depend upon the health of the HCW. Burnout and moral injury prevention support LHS frameworks and core values as they:

- Nurture individual healthcare providers (person‐focused)
- Provide for a diverse workforce with diverse patients (inclusive)
- Allow HCWs to respond to urgent needs (adaptability)
- Provide substance for trust building (governance‐oriented)
- Allow leaders to rise to the top of their capabilities (participatory leadership)
- Allow capacity for incorporating rigorous new knowledge (scientific integrity), and
- Support HCWs in optimizing quality and moderating costs (value).

Given the benefits of preventing burnout and moral injury, both for health systems and for embedded scholars, we pursued “next steps” via an interactive learning session focused on creating a sustainable and ethical work climate within an LHS, with the result being a call for a new competency in moral injury and burnout reduction.

## 4 EDUCATIONAL SESSION TO INCORPORATE THOUGHTS OF LHS SCHOLARS

To bring LHS scholars into the process, we planned a session on burnout and moral injury prevention. Four weeks before the session, we distributed two brief articles^3^, ^11^ and asked scholars to respond to two questions:

1. How does moral injury differ from burnout as a way of describing work distress?
2. How might you organize an LHS project to reduce moral injury?

**FIGURE 2:**
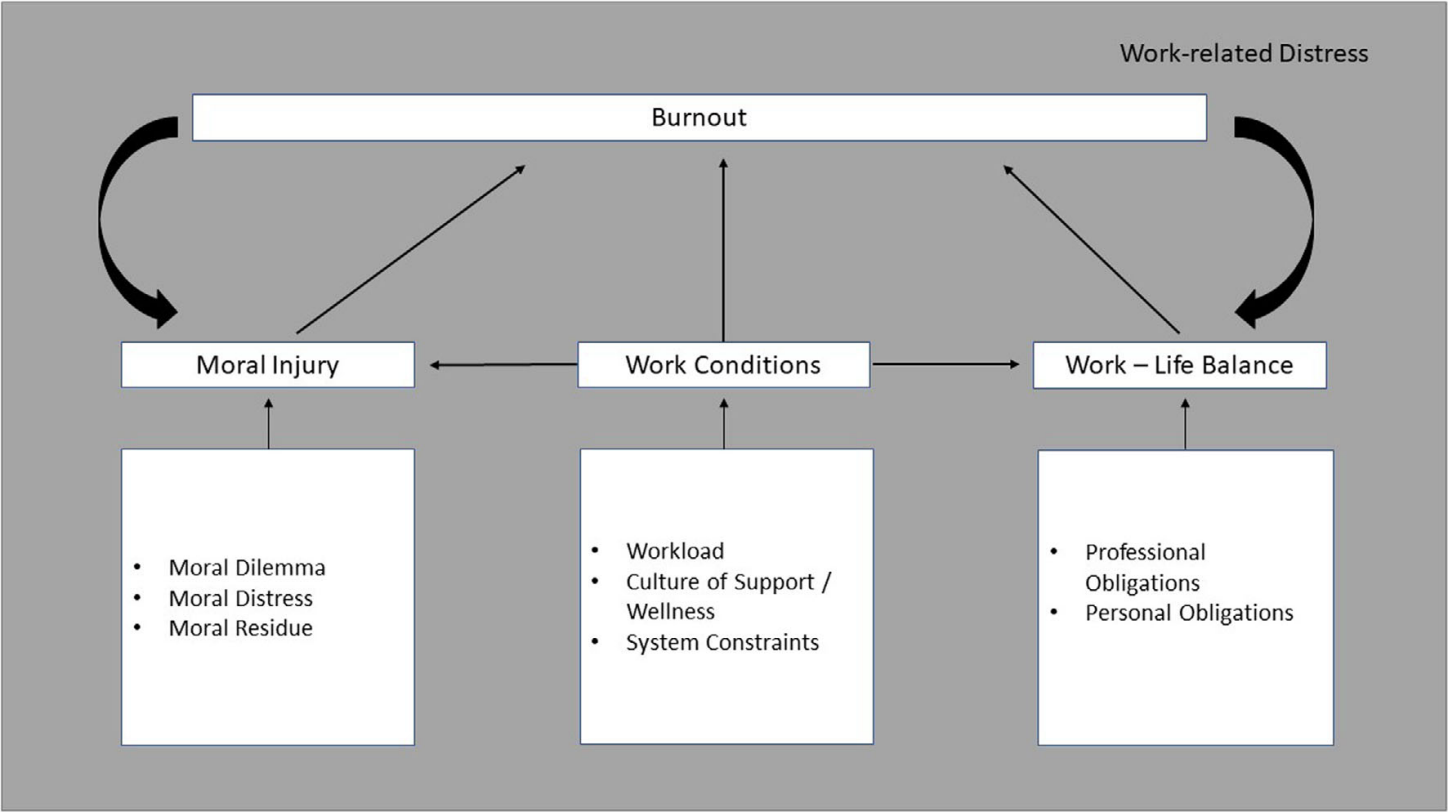
Model linking work conditions, work‐life balance, moral injury, and burnout. Conceptual model by author W.M. linking work conditions, work‐life balance, moral injury, and burnout, demonstrating proposed relationships between these variables and points for interventions to improve the work environment within Learning Health Systems.

*Replies to the prework* were collated, reviewed, and used to prompt discussion during the Design Shop. An informal review of themes from LHS scholars’ comments was later performed by one author (M.L.). The LHS scholars (12 surveyed, 4 (33%) responded (2 females, 2 males, 1 MD, 3 Ph.Ds)) provided written comments that were viewed within the lens of SQUIRE 2.0 guidelines for Quality Improvement reporting, with comments addressing HCW burnout, providing the rationale for existing LHS constructs, elaborating on methods to develop these models, and offering a summary of ways forward to address burnout and moral injury reduction. One model by co‐author and LHS scholar W.M. (Figure 2) presented actionable emphases on work conditions and work‐life balance. Among themes identified, LHS scholars included contrasts of burnout versus moral injury, promising methodologies (such as Ecological Momentary Assessment, known for real‐time assessment of physiologic and psychologic variables,^17^, ^18^ and using a Moral Injury Symptom Scale^19^ to study resident burnout), interventions (including self‐care training and revisions in workflows), and acknowledging unique impacts of COVID‐19. New framing of moral injury etiologies included insufficient time with patients, COVID‐related care rationing, inadequate attention to invisible (noncountable) work, and mismatches between scholars’ values and mandates in how they were to practice medicine or perform scholarship (see Table 1). Ecologic Momentary Assessment, in particular, has been used extensively to study wellbeing, capturing the fluid nature of daily emotions. Across studies, ~72% of assessments were completed by participants, with the best engagement in assessment periods of 1–2 weeks.^20^

**TABLE 1:** Recommendations from LHS Scholars pre‐work and attendees at Minnesota LHS Design Shop session 5‐5‐2022 on “Reducing burnout and moral injury.”

Recommendations
Focus on actionable aspects of moral injury, work conditions, and work‐life balance to reduce burnout
Contrast burnout versus moral injury
Use targeted methods to pursue learning about burnout and moral injury, including
Ecological Momentary Assessment
Asset versus deficit‐based approaches (including appreciative inquiry)
Use of validated instruments, such as the Moral Injury Symptom Scale
Test interventions (self‐care training, workflow redesign) in using QI methods
Develop methods to address unique aspects of workflow surges (due, for example, to COVID‐19)
Catalog and study current morally injurious situations, such as
Insufficient time with patients
COVID‐19‐related care rationing
Lack of attention to “invisible work” by minoritized subgroups
Lack of time for self‐care
Mismatches between scholar values and how (a) clinical scholars are told to practice medicine and (b) nonclinical scholars are told to select and perform scholarship
Relate moral injury prevention to high‐impact topics such as
Promoting equity, professionalism, and injury
The need for “system change” to promote fairness and sustainability

During the Design Shop “virtual session” on 5‐5‐22, with approximately 30 regional participants (4 national and regional experts on moral injury and ethics (two were physicians), nine LHS scholars/trainees (four physicians), nine Minnesota LHS program leaders (two physicians), six colleagues from University of Minnesota's Center for LHS Sciences, two PhD students, two PhD visiting scholars, and one VA fellow), leading‐edge scientific issues arose, including an Asset versus Deficit based approach (assessed via Appreciative Inquiry^21^, ^22^ as a means to learn about successes in creating supportive work climates), the relationship of moral injury to equity (with new instruments to assess negative experiences due to race and gender,^23^), how professionalism challenges lead to injury, and the need for systems change to promote fairness, inclusion, and sustainability (Table 1). We note that appreciative inquiry has been used to create interventions to reduce burnout in nurses.^24^

## 5 A COMPETENCY IN BURNOUT AND MORAL INJURY PREVENTION

Based on these discussions, we have confidence that burnout and moral injury prevention will be relevant locally and nationally.^25^ Burnout and moral injury prevention represent an important educational domain to LHS scholars (Question 1), which can serve to protect LHS scholars and institutional colleagues from burnout and moral injury (Question 2) while providing “value added” by encouraging scholars to include this topic in studies that benefit their institutions (Question 3). In a study in medical faculty in Iran,^26^ burnout related to lower interest in incorporating new curricula; thus, burnout reduction holds promise for facilitating the incorporation of new ideas generated in an LHS. While there is some overlap with health systems research ethics, the science of burnout and moral injury has largely been relegated to an “other” category, even while burnout has skyrocketed.^25^ The absence of a specific competency has been accompanied by a lack of focus in this aspect of LHS science that has consequently not kept pace with the rising burnout rates. We thus describe and issue a call for a new Competency Domain for LHS programs in *burnout and moral injury prevention*:

To ensure clinician and researcher wellness (in this case, prevention of burnout and moral injury) is promoted in clinical practice and academic endeavors.

*Competencies*:

1. Demonstrate ability to assess burnout and moral injury in an organization using standard metrics while identifying remediable predictors of these outcomes.
2. Demonstrate knowledge of components of burnout and moral injury and how to address work climates to become more ethical^27^ by using a Four A approach (Ask, Affirm, Assess, Act).
3. Demonstrate knowledge of promising new methods, including Ecologic Momentary Assessment and appreciative inquiry, while explicitly addressing equity as a component of burnout.
4. Demonstrate ability to structure an LHS training program with a focus on burnout and moral injury prevention leading to enhanced retention.
5. Demonstrate means of embedding burnout/moral injury prevention into an LHS to benefit researchers, HCWs, and patients.

This conceptualization of a burnout/moral injury prevention competency is limited by the development in one setting with a small number of LHS scholars (nine), experts (four) and discussants (approximately thirty). Thus, this work should be viewed as exploratory. We welcome a conversation on burnout and moral injury prevention as we articulate ways for health systems to create better work environments^28^ for their workers, scholars, and patients.

## CONFLICT OF INTEREST STATEMENT

Dr. Linzer is supported through his employer Hennepin Healthcare for burnout reduction studies and training by large health systems (Optum, Essentia, Gillette, California AHEC) and by national organizations (AMA and IHI). His work on this paper was supported by AHRQ. The other authors have no disclosures.
